# New Horizons in the Treatment of Type 1 Diabetes: More Intense Immunosuppression and Beta Cell Replacement

**DOI:** 10.3389/fimmu.2018.01086

**Published:** 2018-05-17

**Authors:** Carlos E. B. Couri, Kelen C. R. Malmegrim, Maria C. Oliveira

**Affiliations:** ^1^Center of Cell-Based Therapy, Regional Blood Center of Ribeirao Preto, Ribeirao Preto Medical School, University of Sao Paulo, Ribeirao Preto, Brazil; ^2^Department of Internal Medicine, Ribeirao Preto Medical School, University of Sao Paulo, Ribeirao Preto, Brazil; ^3^Department of Clinical, Toxicological and Bromotological Analysis, School of Pharmaceutical Sciences of Ribeirao Preto, University of Sao Paulo, Ribeirao Preto, Brazil

**Keywords:** type 1 diabetes, immunotherapy, autologous hematopoietic stem cell transplantation, immunologic reset, autoimmunity, beta cell preservation

## Abstract

Since the discovery of autoimmunity as the main pathophysiologic process involved in type 1 diabetes, many attempts have tried to delay or stop beta cell destruction. Most research protocols in humans have investigated the effects of therapeutic agents targeting specific steps of the autoimmune response. In spite of safety and some degree of beta cell preservation, the clinical impact of such approaches was similar to placebo. Recently, research groups have analyzed the effects of a more intense and wider immunologic approach in newly diagnosed type 1 diabetic individuals with the “immunologic reset,” i.e., high-dose immunosuppression followed by autologous hematopoietic stem cell transplantation. This more aggressive approach has enabled the majority of patients to experience periods of insulin independence in parallel with relevant increments in C-peptide levels during mixed meal tolerance test. However, on long-term follow-up, almost all patients resumed exogenous insulin use, with subsequent decrease in C-peptide levels. This has been at least in part explained by persistence of islet-specific T-cell auto-reactivity. Here, we discuss future steps to induce immune tolerance in individuals with type 1 diabetes, with emphasis on risks and possible benefits of a more intense transplant immunosuppressive regimen, as well as strategies of beta cell replacement not requiring immunomodulation.

## The Complex Autoimmune Repertoire of Type 1 Diabetes (T1D)

Type 1 diabetes is an autoimmune disease characterized by a silent phase of progressive beta cell destruction, followed by a symptomatic phase of hyperglycemia, when great amount (not well defined) of beta cell mass and function has been reduced. This phenomenon occurs in individuals with genetic background exposed to still undefined immunologic triggers (1). The rate of beta cell destruction is not absolutely linear as previously described. The pattern of temporal beta cell loss is typical of relapsing-remitting diseases, as periods of exacerbated beta cell destruction alternate with those of inactivity (1). In addition, subjects who are older at diagnosis present a slower process of autoimmune destruction, and, as a consequence, larger residual beta cell mass (2, 3).

Recent data from the TEDDY (The Environmental Determinants of Diabetes in the Young) trial showed that autoantibodies against islet antigens may occur early in life of a subset of patients. In fact, the age of patients at diagnosis of overt diabetes and the order of antibody positivity depend both on HLA and non-HLA genotypes (4, 5). The exact triggers of autoimmunity against pancreatic islet beta cells have not yet been identified, and much of the immunologic mechanisms involved in this process are still under study. There is a pathological cross talk involving B and T lymphocytes, regulatory cells (B and T reg cells), autoantibodies, monocytes, natural killer cells, cytokines, and the beta cells themselves (1, 6–8). Although there is evidence that CD8+ effector T cells may be directly involved in beta cell death, other cell types most certainly participate in this autoimmune response (8, 9). In this scenario, which are the effects of immune interventions in humans with T1D?

## The Role of Immunointerventions in the Preservation of Beta Cell Mass

Since the discovery of autoimmunity as the key phenomenon leading to T1D, many immunologic interventions have been investigated as strategies to stop beta cell destruction. The Diabetes Control and Complication Trial showed that higher C-peptide levels are associated with lower incidence of diabetic nephropathy, retinopathy, and less episodes of hypoglycemia (10). Preservation of beta cell mass is also associated with less exogenous insulin requirement (3). Autoimmunity in T1D is complex and involves different pathways, connections, organs, and cells. Nevertheless, most research protocols have attempted to hinder beta cell destruction targeting specific molecules or pathways, instead of wider immunosuppressive approaches. The argument is that systemic immunosuppression may expose the patient to undesirable adverse effects. Table 1 summarizes the main outcomes of recent clinical trials on beta cell preservation in patients with T1D (secondary prevention trials).

**Table 1:** Recent secondary prevention trials in individuals with type 1 diabetes and their effect on beta cell preservation.

Immunomodulatory approach	Main target of medication	Follow-up	Effect on C-peptide on time	Comments
Teplizumab (11)	T cell (CD3+)	2 years	Slower decline compared with placebo	At 1 year, 5.3% (11/207) of patients in the full-dose group were free from insulin. In year 2, three of these 11 were still insulin-free

Otelixizumab (12–15)	T cell (CD3+)	4 years	Slower decline in C-peptide with higher doses of otelizumab. No effect with lower doses	The higher the dose of otelizumab, the better the clinical response, but the more the risk of side effects. No patient became insulin-free

ATG + granulokine (16)	T cell (CD4+, CD8+), B cell, T reg	1 year	Slower decline compared with placebo	No differences in insulin dose compared with placebo. No patient insulin-free. Time of disease at randomization: 4 and 24 months

Rituximab (17)	B cell	2 years	Slower decline compared with placebo	Lower insulin requirements in the treated group. No patient insulin-free

Alefacept (18)	T cell (CD4+, CD8+)	2 years	Slower decline compared with placebo	Reduced insulin dose in treated group. No patient insulin-free

Abatacept (19)	T cell (CD80+, CD 86+, CD28+)	3 years	Slower decline compared with placebo	No differences in insulin dose compared with placebo

Autologous mesenchymal stem cell (21)	T cell, T reg	1 year	No change compared with placebo	No differences in insulin dose compared with placebo. Cells were harvested from bone marrow

Autologous T regs (23)	T reg	2 years	No changes in C-peptide along the time compared to baseline	No differences in insulin dose along the time

Chemotherapy followed by autologous hematopoietic stem cell transplantation (26–35)	“Immunologic reset”	Up to 7 years	Increase in C-peptide > 3 y post-transplantation and then returned to baseline levels after 6 years and (compared to baseline)	Around 80% of patients became insulin-free for variable periods. Lack of randomized, parallel, double-blind, placebo-controlled trials. One death occurred in the Polish group. Potential risk of severe side effects

Effector T cells are directly related to beta cell death, and secondary prevention trials with teplizumab (anti-CD3 monoclonal antibody) (11) and otelixizumab (anti-CD3 monoclonal antibody) (12–15) have used these cells as targets to preserve pancreatic function. In these studies, treated patients presented a less accelerated rate of decline in C-peptide levels and also some reduction in daily insulin doses, when compared to non-treated patients, indicating a beneficial effect of these immunomodulatory agents on beta cell preservation. However, less than 5% of individuals experienced periods free from insulin.

The combination of antithymocyte globulin plus granulocyte colony-stimulating factor (G-CSF) has also been investigated, as another attempt to stop T cell auto-reactivity. Individuals up to 2 years after diagnosis of T1D (different from other secondary prevention trials) were included. Along 1 year, the decline in C-peptide levels was slower in treated patients, when compared to the control group, but there was no difference in insulin requirements (16). In 2014, Peskovitz and colleagues turned the focus on B cells, treating newly diagnosed T1D individuals with rituximab (anti-CD20 monoclonal antibody). The treatment was considered safe and able to induce slower decline in C-peptide levels; however, no significant difference in insulin requirements was detected between treated and placebo groups (17).

Later, T cells were again targeted by immunomodulatory approaches with alefacept (LFA3-IgG1 fusion protein that binds CD2) (18) and abatacept (CTLA-4-IgG1 fusion protein that binds CD80/CD86) (19) that induce apoptosis and inhibit activation of T-cells, respectively. Both drugs promoted beta cell preservation, with slower rate of reduction of C-peptide levels and were considered safe. Nevertheless, clinical effects were disappointing, as alefacept promoted only slight reduction of insulin requirements, while abatacept did not change them at all.

Cell-based therapies were also investigated in secondary prevention trials. Multipotent mesenchymal stromal cells (MSCs) are considered safe promising tools to change the natural history of T1D and other immune-mediated diseases. They exhibit immunomodulatory potential, migratory capacity to injured and inflamed areas and can contribute to tissue regeneration [directly or by the secretion of bioactive factors (20)]. In 2015, Carlsson and colleagues analyzed the effect of autologous MSCs in adults with recent-onset T1D. In contrast with non-treated T1D patients, who presented a significant decline in serum C-peptide levels, beta cells were still preserved in the MSC-treated group at 1-year follow-up, with stable levels of C-peptide. The beneficial effect on pancreatic function, however, did not reflect on insulin requirements of the MSC-treated group. No relevant side effects were observed (21).

The pathophysiology of T1D also involves defective function of regulatory T cells (Tregs), and perhaps T effector cells that may be refractory to Treg suppression (22). In 2015, Bluestone and colleagues evaluated the effects of intravenous infusions of expanded autologous polyclonal Tregs in recent-onset T1D patients (22). In this phase I open label study, autologous Treg infusions were safe, but did not change the temporal secretion of C-peptide and exogenous insulin use along 2 years of follow-up. Phase 1/2 trials have also investigated the effects of low-dose exogenous IL-2 on Treg function (23, 24). However, although the studies have detected increments in Treg numbers, the effects on glycemic control have yet to be established.

Most of the research protocols in humans used approaches that aimed to target only some steps of autoimmunity repertoire. Despite safety and some degrees of beta cell preservation, the clinical impact of such agents was similar to the placebo groups. Moreover, most of these immunomodulatory agents were originally developed and/or tested to prevent progression of other autoimmune diseases, and they may not necessarily share similar pathophysiological mechanisms of T1D.

Under the rationale of having a wider approach to the complex immune dysfunctions linked to T1D, in 2007 Voltarelli and colleagues published the first trial analyzing the effect of the “immunologic reset” in newly diagnosed T1D patients. The strategy included ablation of the autorreactive immune system, followed by generation of a new and tolerant system through infusion of autologous stem cells. Hematopoietic stem cells were mobilized with cyclophosphamide (2.0 g/m^2^) and G-CSF (10 µg/kg per day) and then were harvested from the peripheral blood by leukapheresis and cryopreserved. Subsequently, hematopoietic stem cells were injected intravenously after conditioning with cyclophosphamide (200 mg/kg) and rabbit antithymocyte globulin (4.5 mg/kg). In this prospective non-randomized trial, most patients (21 out of 25) became insulin-free after transplant. At 4 years posttransplantation, C-peptide levels were significantly higher than pretransplant levels (25–30). Furthermore, at 7 years, most patients had already resumed exogenous insulin use, but C-peptide levels were still similar to those pretransplantation.

Independent research groups have reproduced the Brazilian transplant protocol with slight modifications to increase efficacy of the procedure (31–34). In the Polish protocol, patients underwent 2 or 3 plasmapheresis sessions before transplant and acarbose was used as maintenance drug (31, 32). In the Mexican protocol, the transplant conditioning regimen included cyclophosphamide plus fludarabine (34). Nevertheless, results regarding duration of insulin independence and transient increase in C-peptide levels were similar to those shown by Voltarelli and colleagues.

Recently, to compare long-term effects of the “immunologic reset” with the real world scenario, a cross-sectional analysis was made with BrazDiab1 (the largest multicenter observational study in T1D in Brazil) data. During the long-term follow-up of 8 years, none of the transplantated patients had developed microvascular complications, while 21.5% of the non-transplanted BrazDiab1 patients had presented at least one microvascular complication (30). Despite limitations, this study suggests that hematopoietic stem cell transplantation may promote long-term beneficial metabolic effects beyond insulin freedom.

Ethical and safety issues are key points of research protocols that involve high-dose systemic immunosuppression. Since there are potential short-term risks of infection, acute organ dysfunction and death, and theoretical long-term risks of malignancies and secondary autoimmune diseases, the inclusion of young children with T1D has been restricted in these trials. The majority of patients included in the studies presented only nausea, vomiting, alopecia, and fever as transplant-related adverse events. In the Polish study, however, there was one death due to *Pseudomonas aeruginosa* sepsis (32). To date, no severe long-term side effects have been described.

In 2017, Malmegrim and colleagues analyzed the effects of autologous hematopoietic stem cell transplantation on the immune system (27). Although CD8^+^ T-cells reconstituted early after transplant, CD4^+^ T-cell remained lower than baseline for several months, resulting in a prolonged inversion of the CD4/CD8 ratio. B cells reconstituted to baseline levels at 2–3 months posttransplantation and regulatory T cell (CD4^+^CD25^hi^FoxP3^+^ and CD8^+^CD28^−^CD57^+^) counts increased. In the overall population, memory cells comprised most of T cells detected on follow-up of patients after transplantation; however, in patients that remained insulin-free for longer periods after transplant, there was slower reconstitution of effector memory cells. When analyzed separately, islet-specific autoreactive CD8^+^ T cells were still present after high-dose immunosuppression, indicating insufficient ablation of these cells.

The lack of knowledge of the exact mechanisms of disease, genetics, and environmental triggers may be one of the reasons for not restoring immunological balance in secondary prevention trials. On the other hand, the organ-specific autoreactivity may be too intense and persistent to be controlled, even by systemic ablation of the immune system.

## The Future: Beta Cell Replacement or More Intense Immunosuppression?

As secondary prevention trials did not achieve complete restoration of immune balance, development of new strategies to preserve and/or increase beta cell mass are still required. If even the most intense immune-based approach of “immunologic reset” with autologous hematopoietic stem cell transplantation was not able to change the natural history of T1D, it is less probable that low intensity or target-specific immunomodulatory approaches would achieve clinical success in this field.

**Figure 1:**
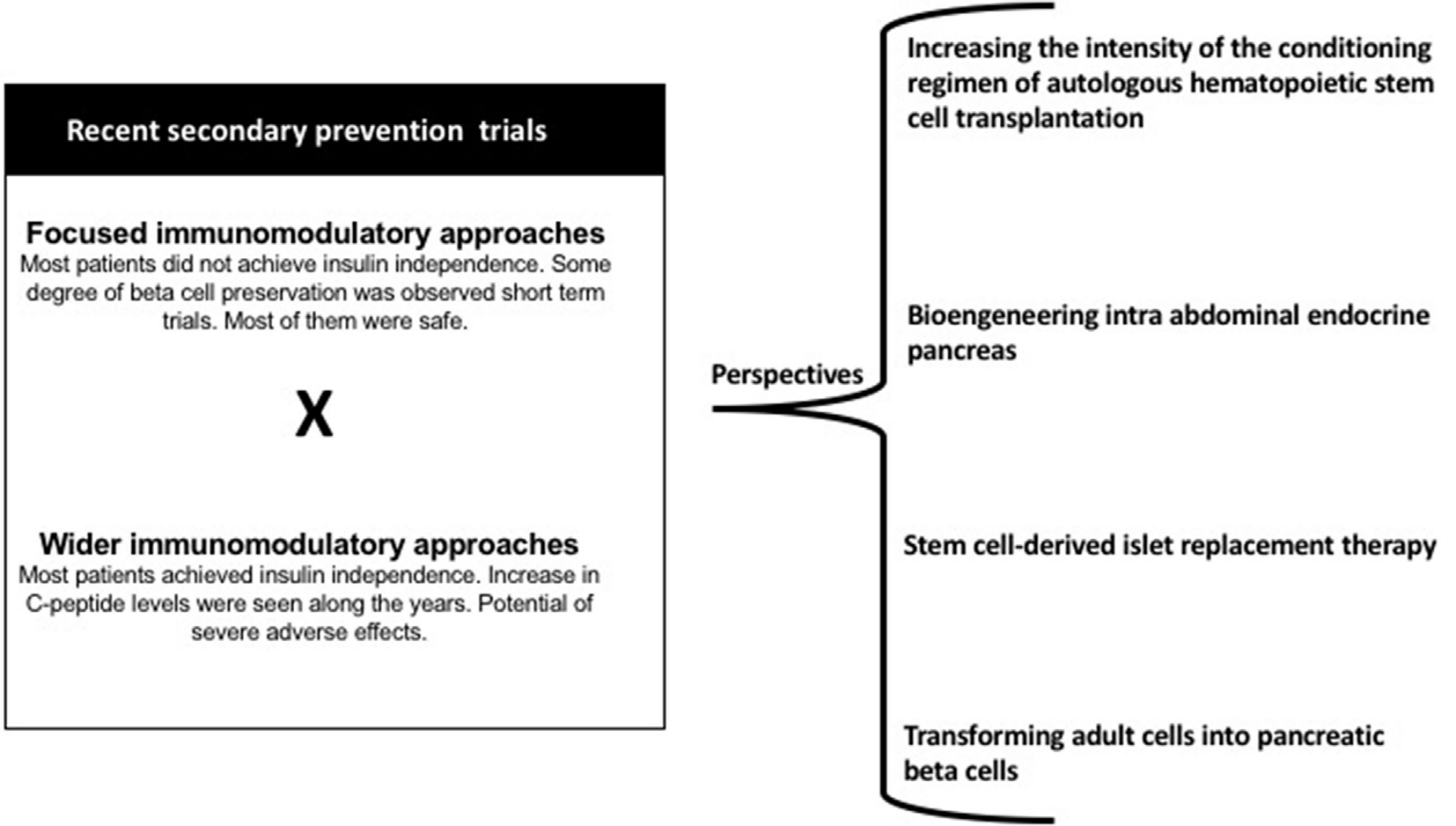
Main results of recent secondary prevention trials and perspectives in the immunologic approaches for individuals with T1D.

Figure 1 presents some of the strategies that can be investigated in the near future to manage T1D autoreactivity.

### Increasing the Intensity of the Conditioning Regimen for Autologous Hematopoietic Stem Cell Transplantation

As previously shown, clinical trials with autologous hematopoietic stem cell transplantation for T1D included similar drugs as conditioning regimen. The Brazilian protocol used high dose cyclophosphamide plus rabbit ATG, while the Polish protocol added plasmapheresis to the procedure and the Mexican protocol used fludarabine plus cyclophosphamide. All provided similar outcomes, indicating insufficient control of islet-specific autoreactivity. Therefore, new protocols of autologous hematopoietic stem cell transplantation should be developed, aiming to increase effectiveness of the immunosuppressive approach. One possibility is to increase the intensity of transplant conditioning regimens. In this context, a three-drug immunosuppressive regimen (cyclophosphamide + fludarabine + rabbit antithymocyte globulin) regimen has been proposed, aiming to more efficiently destroy the memory T and B cell compartment and possibly improve treatment outcomes (28).

Graft manipulation with CD34^+^ may also be investigated as a strategy to be added to future transplant protocols. Although there is no consensus and still a matter of debate in transplantation for other autoimmune diseases, CD34^+^ selection has not been investigated in T1D. The rationale for this approach is that during unselected infusions, memory T cells are reinfused, perpetuating the autoimmune process after transplant. Importantly, graft manipulation is associated with higher incidence of posttransplant viral infections.

For every newly proposed intervention, safety and long-term toxicity must be considered, especially when higher immunosuppression is expected. Despite lifetime insulin-dependence and poor quality of life of patients, T1D is a non-malignant disease, and new strategies to improve glycemic control are constantly under investigation.

In addition to the immune-based interventions, other ongoing research protocols investigate means to restore insulin secretion. Most of the current effort involves development of technologies for beta cell or stem cell encapsulation.

### Intra-Abdominal Endocrine Pancreas Bioengineering

One of the greatest challenges in islet transplantation is the need for chronic immunosuppression (with or without corticosteroids) to avoid rejection of allogeneic cells. This problem may be circumvented by encapsulation of the islet cells to physically prevent host immune cells from reaching the graft.

Recently, Baidal and colleagues (35) reported the case of a woman with longstanding T1D who received pancreatic islets from a deceased donor and became insulin-free 17 days after the procedure. Islets were combined with host autologous plasma and were laparoscopically layered onto the omentum. A degradable biologic scaffold was created, but immunosuppression regimen still had to be used in this case.

The next step will be the improvement of the technique of islet encapsulation so that immunosuppression will be no longer needed. In the future, embryonic stem cells or even induced pluripotent stem cells (iPS) may be used as alternative sources for insulin-producing cells.

### Stem Cell-Derived Islet Replacement Therapy

As a further step in the use of allogeneic insulin-producing cells, an ongoing study investigates transplantation of pancreatic endoderm cells derived from human embryonic stem cells to restore insulin and glucose homeostasis (NCT02239354). Immune-mediated rejection is prevented by a surrounding semi-permeable and protective membrane, enabling pancreatic endoderm cells to further differentiate into mature and functional pancreatic cells, and not requiring use of immunosuppressive drugs.

### Transforming Adult Cells Into Pancreatic Beta Cells

The use of autologous sources for beta cell replacement is another strategy to avoid rejection. *Doiron* and colleagues designed a lentiviral vector construct expressing the glucokinase gene under control of the cytomegalovirus promoter (36). In this study, insulin-secreting cells could be generated, *in vivo*, from adult pancreatic tissue of a mouse model of partial pancreatectomy. Treated animals presented long-term normalization of glucose tolerance and insulin secretion. Despite technical difficulties still to be circumvented, mainly the use of non-viral vectors, this is an attractive approach to restore organ function on humans. As beta cells would be generated from the patient’s own tissue, no immunosuppression would be necessary.

## Conclusion

Many attempts have been made to modulate or even to reset the immune system in type 1 diabetic individuals, aiming to avoid pancreatic beta cell destruction. However, even high-dose immunosuppression followed by infusion of autologous hematopoietic stem cells was not able to sustainedly restore immune tolerance. Given these observations, new approaches need to be developed. These would include the use of more intense immunosuppressive protocols to preserve beta cell mass, perhaps coupled with replacement of beta cells protected against immune destruction.

## Author Contributions

CEBC, KCRM, and MCO researched data and wrote the manuscript.

## Conflict of Interest Statement

The authors declare that the research was conducted in the absence of any commercial or financial relationships that could be construed as a potential conflict of interest.

## References

[1] Atkinson MA Eisenbarth GS Michels AW. Type 1 diabetes. Lancet (2014) 383(9911):69–82. 10.1016/S0140-6736(13)60591-7 23890997 PMC4380133

[2] Pozzilli P Di Mario U Autoimmune diabetes not requiring insulina at diagnosis (latent autoimune diabetes of the adult). Diabetes Care (2001) 24(8):1460–7. 10.2337/diacare.24.8.1460 11473087

[3] Hao W Gitelman S DiMeglio LA Boulware D Greenbaum CJ Type 1 Diabetes TrialNet Study Group. Fall in C-peptide during first 4 years from diagnosis of type 1 diabetes: variable relation to age, HbA1c, and insulin dose. Diabetes Care (2016) 39(10):1664–70. 10.2337/dc16-0360 27422577 PMC5033079

[4] Krischer JP Lynch KF Lernmark Å Hagopian WA Rewers MJ She JX Genetic and environmental interactions modify the risk of diabetes-related autoimmunity by 6 years of age: the TEDDY Study. Diabetes Care (2017) 40(9):1194–202. 10.2337/dc17-0238 28646072 PMC5566280

[5] Krischer JP Liu X Lernmark Å Hagopian WA Rewers MJ She JX The influence of type 1 diabetes genetic susceptibility regions, age, sex, and family history on the progression from multiple autoantibodies to type 1 diabetes: a TEDDY Study Report. Diabetes (2017) 66(12):3122–9. 10.2337/db17-0261 28903990 PMC5697938

[6] Katsarou A Gudbjörnsdottir S Rawshani A Type 1 diabetes mellitus. Nat Rev Dis Primers (2017) 30(3):17016 10.1038/nrdp.2017.16 28358037

[7] Bluestone JA Herold K Eisenbarth G. Genetics, pathogenesis and clinical interventions in type 1 diabetes. Nature (2010) 464(7293):1293–300. 10.1038/nature08933 20432533 PMC4959889

[8] Naushad N Perdigoto AL Rui J Herold KC. Have we pushed the needle for treatment of type 1 diabetes? Curr Opin Immunol (2017) 49:44–50. 10.1016/j.coi.2017.09.004 28992525 PMC5937133

[9] Martin S Wolf-Eichbaum D Duinkerken G Scherbaum WA Kolb H Noordzij JG Development of type 1 diabetes despite severe hereditary B-cell deficiency. N Engl J Med (2001) 345(14):1036–40. 10.1056/NEJMoa010465 11586956

[10] The Diabetes Control and Complications Trial Research Group. Effect of intensive therapy on residual beta-cell function in patients with type 1 diabetes in the diabetes control and complications trial. Ann Intern Med (1998) 128(7):517–23. 10.7326/0003-4819-128-7-199804010-00001 9518395

[11] Hagopian W Ferry RJ Jr Sherry N Carlin D Bonvini E Johnson S Teplizumab preserves C-peptide in recent-onset type 1 diabetes: two-year results from the randomized, placebo-controlled Protégé trial. Diabetes (2013) 62(11):3901–8. 10.2337/db13-0236 23801579 PMC3806608

[12] Guglielmi C Williams SR Del Toro R Pozzilli P. Efficacy and safety of otelixizumab use in new-onset type 1 diabetes mellitus. Expert Opin Biol Ther (2016) 16(6):841–6. 10.1080/14712598.2016.1180363 27145230

[13] Herold KC Hagopian W Auger JA Poumian-Ruiz E Taylor L Donaldson D Anti-CD3 monoclonal antibody in new-onset type 1 diabetes mellitus. N Engl J Med (2002) 346:1692–8. 10.1056/NEJMoa012864 12037148

[14] Keymeulen B Vandemeulebroucke E Ziegler AG Mathieu C Kaufman L Hale G Insulin needs after CD3- antibody therapy in new-onset type 1 diabetes. N Engl J Med (2005) 352:2598–608. 10.1056/NEJMoa043980 15972866

[15] Aronson R Gottlieb PA Christiansen JS Donner TW Bosi E Bode BW Low-dose otelixizumab anti-CD3 monoclonal antibody DEFEND-1 study: results of the randomized phase III study in recent-onset human type 1 diabetes. Diabetes Care (2014) 37(10):2746–54. 10.2337/dc13-0327 25011949 PMC4392937

[16] Haller MJ Gitelman SE Gottlieb PA Michels AW Perry DJ Schultz AR Antithymocyte globulin plus G-CSF combination therapy leads to sustained immunomodulatory and metabolic effects in a subset of responders with established type 1 diabetes. Diabetes (2016) 65(12):3765–75. 10.2337/db16-0823 27669730 PMC5127248

[17] Pescovitz MD Greenbaum CJ Bundy B Becker DJ Gitelman SE Goland R B-lymphocyte depletion with rituximab and β-cell function: two-year results. Diabetes Care (2014) 37(2):453–9. 10.2337/dc13-0626 24026563 PMC3898764

[18] Rigby MR Harris KM Pinckney A DiMeglio LA Rendell MS Felner EI Alefacept provides sustained clinical and immunological effects in new-onset type 1 diabetes patients. J Clin Invest (2015) 125(8):3285–96. 10.1172/JCI81722 26193635 PMC4623571

[19] Orban T Bundy B Becker DJ Dimeglio LA Gitelman SE Goland R Co-stimulation modulation with abatacept in patients with recent-onset type 1 diabetes: follow-up one year after cessation of treatment. Diabetes Care (2014) 37(4):1069–75. 10.2337/dc13-0604 24296850 PMC3964491

[20] Yaochite JN de Lima KW Caliari-Oliveira C Palma PV Couri CE Simões BP Multipotent mesenchymal stromal cells from patients with newly diagnosed type 1 diabetes mellitus exhibit preserved in vitro and in vivo immunomodulatory properties. Stem Cell Res Ther (2016) 7:14. 10.1186/s13287-015-0261-4 26781648 PMC4861132

[21] Carlsson PO Schwarcz E Korsgren O Le Blanc K. Preserved β-cell function in type 1 diabetes by mesenchymal stromal cells. Diabetes (2015) 64(2):587–92. 10.2337/db14-0656 25204974

[22] Bluestone JA Buckner JH Fitch M Gitelman SE Gupta S Hellerstein MK Type 1 diabetes immunotherapy using polyclonal regulatory T cells. Sci Transl Med (2015) 7(315):315ra189. 10.1126/scitranslmed.aad4134 26606968 PMC4729454

[23] Hartemann A Bensimon G Payan CA Jacqueminet S Bourron O Nicolas N Low-dose interleukin 2 in patients with type 1 diabetes: a phase 1/2 randomised, double-blind, placebo-controlled trial. Lancet Diabetes Endocrinol (2013) 1:295–305. 10.1016/S2213-8587(13)70113-X 24622415

[24] Todd JA Evangelou M Cutler AJ Pekalski ML Walker NM Stevens HE Regulatory T cell responses in participants with type 1 diabetes after a single dose of interleukin-2: a non-randomised, open label, adaptive dose-finding trial. PLoS Med (2016) 13(10):e1002139 10.1371/journal.pmed.1002139 27727279 PMC5058548

[25] Voltarelli JC Couri CE Stracieri AB Oliveira MC Moraes DA Pieroni F Autologous nonmyeloablative hematopoietic stem cell transplantation in newly diagnosed type 1 diabetes mellitus. JAMA (2007) 297(14):1568–76. 10.1001/jama.297.14.1568 17426276

[26] Couri CE Oliveira MC Stracieri AB Moraes DA Pieroni F Barros GM C-peptide levels and insulin independence following autologous nonmyeloablative hematopoietic stem cell transplantation in newly diagnosed type 1 diabetes mellitus. JAMA (2009) 301(15):1573–9. 10.1001/jama.2009.470 19366777

[27] Malmegrim KC de Azevedo JT Arruda LC Abreu JR Couri CE de Oliveira GL Immunological balance is associated with clinical outcome after autologous hematopoietic stem cell transplantation in type 1 diabetes. Front Immunol (2017) 8:167 10.3389/fimmu.2017.00167 28275376 PMC5319960

[28] de Oliveira GL Malmegrim KC Ferreira AF Tognon R Kashima S Couri CE Up-regulation of fas and fasL pro-apoptotic genes expression in type 1 diabetes patients after autologous haematopoietic stem cell transplantation. Clin Exp Immunol (2012) 168(3):291–302. 10.1111/j.1365-2249.2012.04583.x 22519592 PMC3390481

[29] Couri CE de Oliveira MC Simões BP. Risks, benefits, and therapeutic potential of hematopoietic stem cell transplantation for autoimmune diabetes. Curr Diab Rep (2012) 12(5):604–11. 10.1007/s11892-012-0309-0 22864730

[30] Penaforte-Saboia JG Montenegro RM Jr Couri CE Batista LA Montenegro APDR Fernandes VO Microvascular complications in type 1 diabetes: a comparative analysis of patients treated with autologous nonmyeloablative hematopoietic stem-cell transplantation and conventional medical therapy. Front Endocrinol (2017) 8:331. 10.3389/fendo.2017.00331 29218029 PMC5703738

[31] Snarski E Milczarczyk A Torosian T Paluszewska M Urbanowska E Król M Independence of exogenous insulin following immunoablation and stem cell reconstitution in newly diagnosed diabetes type I. Bone Marrow Transplant (2011) 46:562–6. 10.1038/bmt.2010.147 20581881

[32] Snarski E Milczarczyk A Hałaburda K Torosian T Paluszewska M Urbanowska E Immunoablation and autologous hematopoietic stem cell transplantation in the treatment of new-onset type 1 diabetes mellitus: long-term observations. Bone Marrow Transplant (2016) 51(3):398–402. 10.1038/bmt.2015.294 26642342

[33] Li L Shen S Ouyang J Hu Y Hu L Cui W Autologous hematopoietic stem cell trans-plantation modulates immunocompetent cells and improves b-cell function in Chinese patients with new onset of type 1 diabetes. J Clin Endocrinol Metab (2012) 97:1729–36. 10.1210/jc.2011-2188 22419704

[34] Cantú-Rodríguez OG Lavalle-González F Herrera-Rojas MÁ Jaime-Pérez JC Hawing-Zárate JÁ Gutiérrez-Aguirre CH Long-term insulin independence in type 1 diabetes mellitus using a simplified autologous stem cell transplant. J Clin Endocrinol Metab (2016) 101(5):2141–8. 10.1210/jc.2015-2776 26859103

[35] Baidal DA Ricordi C Berman DM Alvarez A Padilla N Ciancio G Bioengineering of an intraabdominal endocrine pancreas. N Engl J Med (2017) 376(19):1887–9. 10.1056/NEJMc1613959 28489987 PMC5572072

[36] Doiron B Hu W DeFronzo RA. Beta cell formation in vivo through cellular networking, integration and processing (CNIP) in wild type adult mice. Curr Pharm Biotechnol (2016) 17(4):376–88. 10.2174/1389201017666151223124031 26696016 PMC5421132

